# Malignant Bowel Obstruction: A Retrospective Multicenter Cohort Study

**DOI:** 10.3390/jcm13010263

**Published:** 2024-01-02

**Authors:** Maria-Evanthia Sotirianakou, Maximos Frountzas, Athina Sotirianakou, Haridimos Markogiannakis, George E. Theodoropoulos, Sotirios Sotirianakos, Konstantinos G. Toutouzas

**Affiliations:** 1 First Propaedeutic Department of Surgery, Hippocration General Hospital, School of Medicine, National and Kapodistrian University of Athens, 11528 Athens, Greece; anthi.sotirianakou@gmail.com (M.-E.S.); markogiannakis@yahoo.com (H.M.); tousur@hotmail.com (K.G.T.); 2 Second Department of Surgery, Aretaieion Hospital, School of Medicine, National and Kapodistrian University of Athens, 11528 Athens, Greece; astrnkou@gmail.com; 3 Surgery Department, Sparta General Hospital, 23100 Sparta, Greece; sotksotir@gmail.com

**Keywords:** cancer, bowel, obstruction, ileus, malignant

## Abstract

Background: Malignant bowel obstruction (MBO) is a serious clinical entity that requires surgical intervention in almost 50% of cases. However, overall survival remains low even for operable cases. The aim of the present study was to investigate the correlation between patients’ characteristics, perioperative details, histopathological results and postoperative outcomes of patients who were operated on due to MBO. Methods: A retrospective search of patients who were operated on due to MBO in a university and a rural hospital was conducted. Patients’ characteristics, perioperative details, histopathological results and postoperative outcomes were reported. Univariable and multivariable analysis was performed. Results: Seventy patients were included with a mean age of 76.1 ± 10.6 years. The 30-day mortality rate was 18.6%, the Intensive Care Unit (ICU) admission rate was 17.1% and the mean length of stay (LOS) was 12.4 ± 5.7 days. Postoperative 30-day mortality was associated with increased age, known malignant recurrence, microscopically visible metastatic foci and defunctioning stoma creation. Colorectal malignancy type, sigmoid obstruction and primary anastomosis were correlated with decreased 30-day mortality. In addition, operation at the university hospital led to increased LOS, while stoma creation led to decreased LOS. Finally, ICU admission rates were increased for operations at university hospitals, at least one comorbidity, known malignant recurrence and longer preoperative waiting interval, whereas they were decreased for colorectal primary malignancy type. Conclusions: Surgery due to MBO leads to increased morbidity and mortality. Therefore, prospective studies are needed to highlight inter-patient differences regarding the best individualized therapeutic strategy.

## 1. Introduction

Colorectal cancer is the most common type of cancer of the gastrointestinal tract [1]. Despite the large amount of available data, the increased awareness of screening and the improvement of treatment methods, the initial presentation of stage 4 colorectal cancer cases is not rare [2]. A serious manifestation of advanced disease is malignant bowel obstruction (MBO), which is the most common indication for palliative surgical consultation [3]. The definition of malignant bowel obstruction has not yet been completely clarified, but the most widely accepted criteria are (a) clinical evidence of bowel obstruction, physical examination, or radiographic examination, (b) obstruction distal to the Treitz ligament, (c) primary intra-abdominal incurable cancer, or (d) extra-abdominal primary cancer with peritoneal involvement [4].

Between 10 and 28% of patients with colorectal cancer and up to 50% of patients with ovarian cancer will develop malignant bowel obstruction at some point in their lives [5]. While most people with malignant bowel obstruction have distant metastases, 10% present an isolated metastatic lesion resulting in obstruction [6]. Operable disease has a survival of 3–8 months, whereas inoperable cases present a survival of 4–5 weeks [7]. Although ideal therapeutic management is still under controversy, surgical intervention could not be avoided in almost 50% of cases worldwide [8].

The aim of the present study was to investigate possible correlations between the preoperative characteristics of patients presenting with malignant bowel obstruction, as well as perioperative parameters and their histopathological reports with their postoperative outcomes, such as hospitalization interval, ICU admission and postoperative mortality.

## 2. Material and Methods

### 2.1. Study Design and Participants

The present study included retrospectively enrolled patients who underwent emergent surgery due to malignant bowel obstruction at the ‘’Hippocration’’ General Hospital of Athens and the General Hospital of Sparta from 2017 until 2021. Inclusion criteria were age 18–90 years old, emergency admissions, urgent or emergent operations, bowel obstruction based on Computed Tomography (CT) with oral contrast, obstructing point at small or large intestine and preoperative or intraoperative diagnosis of gastrointestinal or other malignancy. Exclusion criteria were age below 18 or above 90 years old, inflammatory diseases leading to intestinal obstruction, bowel perforation and re-operations due to postoperative ileus.

### 2.2. Ethical Approvals

This study was approved by the Ethical Committees of ‘’Hippocration’’ General Hospital of Athens and General Hospital of Sparta. It was conducted in compliance with the Declaration of Helsinki guidelines about ethical principles for medical research involving human subjects. A written informed consent was obtained by all patients before participation in the study. The present manuscript has been prepared according to the Committee on Publication Ethics (COPE) guidelines.

### 2.3. Study Outcomes

A retrospective search of included patients’ medical records was conducted after institutional approvals were obtained. Patient demographic data (age, gender, medical history, surgical history, type of hospital), patient information at the time of diagnosis (malignant disease status, location of obstruction, malignancy origin), operative details, intraoperative details and postoperative outcomes (ICU admission, length of stay, reoperation, 30-day mortality) were reported. In addition, histopathological analysis of specimens was conducted by two histopathologists independently and included information about the type of malignancy, its differentiation, the number of harvested lymph nodes and their infiltration rate, the surgical margins, Tumor Node Metastasis (TNM) staging, as well as perineural and perivascular infiltration. Postoperative mortality at 30 days was predefined as the main study outcome, whereas ICU admission and length of stay were considered as secondary outcomes.

### 2.4. Statistical Analysis

A power analysis was performed considering a power level of 80% and a significance level (alpha) of 0.05. The minimum sample size was 52 patients.

The Kolmogorov–Smirnov test was used to check the normality of distributions among quantitative variables. Mean values and standard deviations (SD) were used for normally distributed outcomes, while medians and interquartile ranges were used for abnormally distributed outcomes. Absolute (N) and relative (%) frequencies were used to describe qualitative variables. Comparisons of proportions were performed with *Pearson’s χ^2^ test* or *Fisher’s exact test*. Comparisons of quantitative variables between the two groups were conducted with the *Student’s t-test* or the non-parametric *Mann*–*Whitney U test*. Correlations between two quantitative variables were performed by the *Pearson* or *Spearman correlation coefficient*.

Stepwise linear regression analysis was conducted to investigate independent factors associated with length of stay, retrieving dependence coefficients (β) and their standard errors (SE). Independent factors related to ICU admission and postoperative 30-day mortality were investigated by logistic regression analyses that were performed with the stepwise inclusion/exclusion procedure and odds ratios along with their 95% confidence intervals (95% CI) were calculated. Significance levels were two-sided and the statistical significance level was set at *p* = 0.05. The statistical program SPSS 22.0 was utilized for the analysis.

## 3. Results

The present study included 70 patients with a mean age of 76.1 ± 10.6 years. Thirty-six (51.4%) patients were male and 34 (48.6%) female. Thirty-one (44.3%) patients were operated at a university hospital, and 39 patients (55.7%) were operated at a rural hospital (Table 1). Twenty-six patients (37.1%) had hypertension, 14 patients (20%) had coronary disease, 12 patients (17.1%) had hypothyroidism and several comorbidities (dyslipidemia, heart arrhythmia, diabetes, COPD, etc.) followed in frequency (Supplementary Table S1). Nine patients (12.9%) reported an appendectomy in their past surgical history, seven patients (10%) had undergone inguinal hernia repair, six patients (8.6%) had a laparoscopic cholecystectomy, four patients (5.7%) had uterus resection and other past surgeries included hip arthroplasty, hemicolectomies, intestinal resections, splenectomy, etc. (Supplementary Table S2).

Seventeen patients (24.3%) presented with a recurrence of an already known malignancy, while for 53 patients (75.7%) the diagnosis of malignancy was initial. Twelve patients (17.1%) had an already known metastatic lesion at the obstruction point and in 34 patients (54%) macroscopic metastatic foci were observed. The mean waiting interval from presentation until operation was 2.8 ± 3 days. Thirty-five patients (50%) underwent colectomy with primary anastomosis, 18 patients (25.7%) underwent Hartmann’s procedure, in 15 patients (21.4%) a defunctioning stoma was created, 8 patients (11.4%) underwent small intestinal resection and in 6 patients (8.6%) other organs (apart from the small intestine, colon and rectum) were resected (Table 1).

The histopathological assessment demonstrated that the majority of patients suffered from colon adenocarcinoma (89.4%) and only five patients (7.5%) suffered from malignancies other than intestinal (two gastric cancers, two pancreatic cancers and one lymphoma). Twenty-one tumors (43.8%) showed low differentiation and 24 tumors (50%) showed moderate differentiation. In 44 patients (89.8%) resection margins were clear and in 41 patients (85.4%) perineural or perivascular infiltrations were present (Table 2). Moreover, the majority of tumors were T3 (47.8%) and T4 (43.5%), while 20 tumors (44.4%) were N0, 11 tumors (24.4%) were N1 and 12 tumors (26.7%) were N2. The mean number of harvested lymph nodes was 18.6 ± 9.7 and the mean number of infiltrated lymph nodes was 2.9 ± 5.3. Finally, 24 tumors (60%) were metastatic (Supplementary Table S3).

The majority of tumors originated from the sigmoid colon (42.9%), 15 tumors (21.4%) were located at the rectum (21.4%), 9 tumors (12.9%) at the ascending colon, 5 (7.1%) at the cecum, 5 (7.1%) at the transverse colon, 5 (7.1%) at the descending colon, 2 (2.9%) at the small intestine and 5 (7.1%) in organs different from the intestine (Supplementary Table S4). In addition, 12 patients (17.1%) were admitted to the ICU and 3 patients (4.3%) were re-operated. Two patients were re-operated due to wound dehiscence and one patient was re-operated due to stoma necrosis and wound dehiscence (Table 2). The 30-day mortality rate was 18.6% and the mean length of hospital stay was 12.4 ± 5.7 days (Table 2).

### 3.1. 30-Day Mortality

Univariable analysis of postoperative outcomes revealed that 30-day mortality was associated with increased age (83.5 ± 10.9 years vs. 74,4 ± 9,9 years, *p* = 0.005). Moreover, patients with malignancy recurrence had a greater 30-day mortality rate (47.1% vs. 9.4%, *p* = 0.002). Similarly, a previously known metastatic lesion at the obstruction site was associated with an increased risk for 30-day mortality (40.9% vs. 8.3%, *p* = 0.002). Furthermore, 30-day mortality was greater in patients with macroscopically visible metastatic foci (29.4% vs. 6.9%, *p* = 0.023). On the other hand, 30-day mortality was lower in patients with colorectal cancer type (*p* = 0.034), especially with an obstruction point located at the sigmoid colon (*p* = 0.045). Primary colorectal anastomosis was associated with a lower 30-day mortality rate (*p* = 0.006) and defunctioning stoma creation was correlated to higher 30-day mortality (*p* < 0.001) (Table 3).

### 3.2. Length of Stay

Operation at a university hospital was associated with increased length of stay (13.6 ± 5.1 days vs. 11.4 ± 6.1 days, *p* = 0.042). However, defunctioning stoma creation was associated with decreased length of stay (10,2 ± 6,7 days vs. 13 ± 5,4 days, *p* = 0.04). Finally, preoperative waiting interval was associated with overall length of stay (correlation coefficient 0.5, *p* < 0.001) (Table 4).

### 3.3. ICU Admission

Operation at a university hospital (38.7% vs. 0%, *p* < 0.001) and the presence of at least one comorbidity (31.6% vs. 0%, *p* < 0.001) were associated with increased ICU admission rate (Table 5). Recurrent metastatic disease was also associated with an increased ICU admission rate (41.2% vs. 9.4%, *p* = 0.006), as well as an already known metastatic lesion at the obstruction site (36.4% vs. 8.3%, *p* = 0.007) (Table 5). On the other hand, colorectal type of cancer was associated with a lower ICU admission rate (12.9% vs. 50%, *p* = 0.025). Finally, a longer preoperative waiting interval was associated with a greater risk of ICU admission (5.1 ± 3.5 days vs. 2.4 ± 2.7 days, *p* = 0.002) (Table 5).

### 3.4. Multivariable Analysis

Multivariable analysis demonstrated that 30-day mortality was associated with age (OR 1.21, 95% CI 1.07–1.37, *p* = 0.002) and macroscopically visible metastatic foci (OR 49.61, 95% CI 3.25–758.06, *p* = 0.005) (Table 6). In addition, the creation of a defunctioning stoma was associated with a shorter length of stay (*p* = 0.004). Finally, the initial diagnosis of malignancy was associated with a lower ICU admission rate (OR 0.15, 95% CI 0.04–0.57, *p* = 0.005) (Table 6).

## 4. Discussion

Malignant bowel obstruction (MBO) seems to remain an important healthcare issue, despite the increased screening programs for healthy individuals and meticulous follow-up protocols for already diagnosed cancer patients [9]. The present retrospective study which included patients who were operated on due to malignant intestinal obstruction demonstrated a significant correlation between increased 30-day mortality and increased age, already known recurrence during oncologic follow-up, already known metastatic lesion at the site of obstruction, macroscopically visible metastatic foci intraoperatively and defunctioning stoma creation. On the other hand, lower 30-day mortality rates were observed in patients with colorectal cancer compared to other malignancy types, in patients who presented an obstruction point at the sigmoid colon and in patients who underwent a primary anastomosis. In addition, an operation at a university hospital was associated with an increased length of stay, while defunctioning stoma creation was associated with a decreased length of stay. Finally, increased ICU admission rate was correlated with operation at a university hospital, presence of at least patient comorbidity, already known recurrence during oncologic follow-up, already known metastatic lesion at the site of obstruction and longer preoperative waiting interval, whereas colorectal malignancy was associated with lower ICU admission rate.

Length of hospital stay due to malignant bowel obstruction has been described as about 7 days for conservative treatment, while it significantly increases (mean 11 days, range 6–18 days) for surgical management [10]. In our study, the length of stay was 12.4 ± 5.7 days, which is in concordance with international data. Nevertheless, the length of stay was significantly different between operations at a university (13.6 ± 5.1 days) and a rural hospital (11.4 ± 6.1, *p* = 0.042). A multidisciplinary preoperative approach examining all treatment options, including non-operative ones, such as stenting or medical treatment, which are available in a university hospital could be a reason [11]. In addition, preoperative resuscitation based on ICU protocols that would be available in university hospitals could lead to a longer hospital stay [12]. Nevertheless, longer preoperative waiting interval was correlated to increased ICU admission rates (5.1 ± 3.5 days vs. 2.4 ± 2.7 days, *p* = 0.002) indicating the severity of such cases and the meticulous preoperative preparation that is necessary.

Intra-abdominal “oncologic load”, which is expressed by an already known malignancy recurrence during oncologic follow-up or the presence of macroscopically visible metastatic foci intraoperatively, seems to significantly affect the mortality and morbidity of patients undergoing surgery due to malignant bowel obstruction. Our study indicated that already known recurrences, especially at the site of obstruction, and visible metastatic lesions during operation led to increased 30-day mortality and ICU admission rate. However, a 30-day mortality of 15.7% and an ICU admission rate of 16.3% is reported worldwide [13], which is in concordance with our results (18.6% and 17.1%, respectively). On the other hand, colorectal type as the primary malignancy seemed to be associated with decreased 30-day mortality and ICU admission rates compared to other malignancies, like gastric or pancreatic cancer. Nevertheless, a malignant intestinal obstruction with or without contemporary distant metastases, results in a 5-year survival of 10% and 40.9%, respectively [14].

Making the decision for surgical intervention in a patient with malignant intestinal obstruction is a multifactor procedure, during which a surgeon should examine the availability of alternative means of treatment, the nutritional condition of the patient, the preoperative clinical staging including features of excess “oncologic load” (ascites, extended omental infiltrations) and the possibility of urgent complications such as volvulus, ischemia and perforation [15]. However, when the decision for surgical intervention is made, principles of surgical oncology should be followed when the patients’ condition allows it and the operation could have a curative intent potential. The total number of lymph nodes in the specimen has been associated with overall survival and a number of at least 12 lymph nodes are necessary in order to achieve a precise N-status classification [16]. In addition, a distance of at least 5–7 cm on either side of the tumor is recommended. Infiltrated surgical margins entail an increased risk of local recurrence, occurrence of late metastases, and reduced overall and disease-free survival [17]. In the present study, the negative resection margin rate was 89.8% and the mean number of harvested lymph nodes was 18.6 ± 9.7, which provided an accurate N-stage histopathological assessment (N0 44.4%, N1 24.4%, N2 26.7% and the mean number of infiltrated lymph nodes 2.9 ± 5.3).

The medical condition of patients suffering from malignant intestinal obstruction is usually burdened due to the emergent situation of bowel obstruction and the advanced stage of their malignancy [18]. Consequently, the decision for surgical intervention should be made meticulously, based on the operability of the disease. A curative intent surgical operation should be considered in patients with operable intra-abdominal disease, whereas a quick bypass of the obstruction or a defunctioning stoma creation should be followed in extended-malignancy cases [19]. Nevertheless, our study indicated that a stoma creation was associated with increased 30-day mortality (*p* < 0.001), indicating severely ill patients that undergo a stoma creation. Therefore, non-surgical treatment options should be considered in such cases. The only randomized trial which is available by Krouse et al. demonstrated no difference in terms of good days out of hospital in patients with small bowel malignant obstruction who underwent surgical therapy compared to patients who received non-surgical management both for randomized and patient-choice groups [20]. Endoscopic stent placement shows a high rate of symptom relief (64–100%), but there is a risk for perforation (0–15%), stent migration (0–40%) and re-occlusion (0–33%) [21]. Medical options include anti-secretory agents (somatostatin analog, steroids, scopolamine), pain medications (morphine) and antiemetic therapy (haloperidol, prochlorperazine) target against symptoms with controversial outcomes [22]. Under these circumstances, large prospectively designed clinical trials should be conducted in order to outline the potential benefits of surgical treatment and identify the patient groups that would benefit more.

The present retrospective study focuses on the surgical management and postoperative outcomes of patients with malignant bowel obstruction (MBO) due to several primary cancer types. To the best of our knowledge, this is the first study that includes patients with MBO due to several primary cancers and investigates their impact on postoperative outcomes. In addition, the predefined meticulous scientific design and the variety of investigated parameters were two advantages of the present study. On the other hand, its retrospective nature is an important disadvantage of this study. Another limitation could be its small sample size (n = 70). However, power analysis demonstrated that the minimum sample size was 52 patients. Therefore, the risk for type-I and type-II errors has been diminished.

## 5. Conclusions

Malignant intestinal obstruction is a serious clinical entity that usually needs surgical intervention, which is accompanied by high rates of postoperative mortality and morbidity. Therefore, a meticulous preoperative assessment considering the risks of intervention, the ideal preoperative waiting time and the operative strategy is indicated. Factors such as age, comorbidities, “oncologic load” and primary malignancy type, which affect postoperative course should also be taken into account. In advanced staged cases a multidisciplinary approach considering non-surgical treatment options should be followed. However, large prospective studies should be designed in order to highlight the benefits of different treatment options for different groups of patients.

## Figures and Tables

**Table 1:** Characteristics of included patients who underwent surgical operation due to malignant bowel obstruction.

		Ν	%
**Gender**	Male	36	51.4
	Female	34	48.6
**Type of hospital**	University	31	44.3
	Rural	39	55.7
**Malignancy status**	Recurrence	17	24.3
	Initial presentation	53	75.7
**Previously known metastatic lesion at obstruction site**	Yes	12	17.1
	No	58	82.9
**Macroscopically visible foci**	Yes	34	54.0
	No	29	46.0
**Type of operation**	Colectomy with primary anastomosis	35	50.0
	Hartmann’s procedure	18	25.7
	Stoma	15	21.4
	Intestinal resection	8	11.4
	Non-GI tract organ excision	6	8.6
**Age** **(years), mean (SD)**		76.1 (10.6)	
**Preoperative waiting interval (days), mean (SD)**		2.8 (3.0)	

**Table 2:** The postoperative outcomes of patients that underwent surgery due to malignant bowel obstruction.

		Ν	%
**ICU admission**	No	58	82.9
	Yes	12	17.1
**Re-operation**	Yes	3	4.3
	No	67	95.7
**Reasons for re-operation**	Wound dehiscence	3	4.3
	Stoma necrosis	1	1.4
**30-day Mortality**	No	57	81.4
	Yes	13	18.6
**Length of stay (days), mean (SD)**		12.4 (5.7)	

ICU, Intensive Care Unit.

**Table 3:** Univariable analysis of patients’ characteristics, perioperative details and histopathological assessment with 30-day mortality after malignant bowel obstruction surgery.

		30-Day Mortality				*p*-Value
		No		Yes		
		N	%	N	%	
**Type of hospital**	University	23	74.2	8	25.8	0.165
	Rural	34	87.2	5	12.8	
**Age, mean (SD)**		74.4 (9.9)		83.5 (10.9)		**0.005**
**Gender**	Male	32	88.9	4	11.1	0.099
	Female	25	73.5	9	26.5	
**Comorbidities**	No	30	78.9	8	21.1	0.561
	Yes	27	84.4	5	15.6	
**Previous surgery**	No	42	85.7	7	14.3	0.188
	Yes	15	71.4	6	28.6	
**Malignancy status**	Recurrence	9	52.9	8	47.1	**0.002**
	Initial presentation	48	90.6	5	9.4	
**Previously known metastatic lesion at obstruction site**	Yes	13	59.1	9	40.9	**0.002**
	No	44	91.7	4	8.3	
**Macroscopically visible foci**	Yes	24	70.6	10	29.4	**0.023**
	No	27	93.1	2	6.9	
**Differentiation**	Low	20	95.2	1	4.8	0.369
	Moderate/High	23	85.2	4	14.8	
**No of harvested lymph nodes, mean (SD)**		18.8 (9.9)		14 (4.6)		0.491
**No of infiltrated lymph nodes, mean (SD)**		2.9 (5.4)		2.3 (2.1)		0.791
**Perineural or perivascular infiltrations**	Yes	38	92.7	3	7.3	0.480
	No	6	85.7	1	14.3	
**Τ**	Τis/T1/T2/T3	25	96.2	1	3.8	1.000
	T4	19	95.0	1	5.0	
**Ν status, mean (SD)**		0.9 (0.9)		1 (1.4)		0.906
**Μ**	No	16	100.0	0	0.0	0.136
	Yes	20	83.3	4	16.7	
**Malignancy type: Colorectal cancer**	No	4	50.0	4	50.0	**0.034**
	Yes	53	85.5	9	14.5	
**Malignancy origin: Cecum**	No	52	80.0	13	20.0	0.576
	Yes	5	100.0	0	0.0	
**Malignancy origin: Ascending colon**	No	51	83.6	10	16.4	0.353
	Yes	6	66.7	3	33.3	
**Malignancy origin: Rectum**	No	44	80.0	11	20.0	0.720
	Yes	13	86.7	2	13.3	
**Obstruction point: Ascending colon**	No	49	83.1	10	16.9	0.416
	Yes	8	72.7	3	27.3	
**Obstruction point: Sigmoid colon**	No	31	73.8	11	26.2	**0.045**
	Yes	26	92.9	2	7.1	
**Obstruction point: Rectum**	No	42	77.8	12	22.2	0.272
	Yes	15	93.8	1	6.3	
**Preoperative waiting interval (days), mean (SD)**		2.6 (2.8)		3.8 (3.7)		0.121
**Operation: Colectomy + anastomosis**	No	24	68.6	11	31.4	**0.006**
	Yes	33	94.3	2	5.7	
**Operation: Hartmann’s procedure**	No	40	76.9	12	23.1	0.160
	Yes	17	94.4	1	5.6	
**Operation: Stoma**	No	51	92.7	4	7.3	**<0.001**
	Yes	6	40.0	9	60.0	
**Operation: Intestinal resection**	No	52	83.9	10	16.1	0.161
	Yes	5	62.5	3	37.5	

The significant *p*-values are in bold. T, tumor; M, metastasis.

**Table 4:** Univariable analysis of patients’ characteristics, perioperative details and histopathological assessment with length of hospital stay after malignant bowel obstruction surgery.

		Length of Stay (days), Mean (SD)	*p*-Value
**Type of hospital**	University	13.6 (5.1)	**0.042**
	Rural	11.4 (6.1)	
**Gender**	Male	13.2 (5.9)	0.281
	Female	11.5 (5.4)	
**Comorbidities**	No	12.8 (5)	0.332
	Yes	11.9 (6.5)	
**Previous surgery**	No	11.8 (4.8)	0.484
	Yes	13.9 (7.3)	
**Malignancy status**	Recurrence	13.4 (6.2)	0.555
	Initial presentation	12.1 (5.6)	
**Previously known metastatic lesion at obstruction site**	Yes	13.5 (5.9)	0.378
	No	11.9 (5.6)	
**Macroscopically visible foci**	Yes	12.7 (6.2)	0.451
	No	11.8 (5.6)	
**Differentiation**	Low	11.8 (4.1)	0.778
	Moderate/High	12.6 (7)	
**Perineural or perivascular infiltrations**	Yes	11.8 (4.4)	0.212
	No	13.4 (3.3)	
**Τ**	Τis/T1/T2/T3	12.3 (6)	0.368
	T4	12.9 (5)	
**Μ**	No	12.9 (6.7)	0.934
	Yes	12.6 (6.3)	
**Malignancy type: Colorectal cancer**	No	15.3 (5.1)	0.066
	Yes	12 (5.7)	
**Malignancy origin: Cecum**	No	12.3 (5)	0.248
	Yes	13.4 (12.6)	
**Malignancy origin: Ascending colon**	No	12.4 (6)	0.902
	Yes	12 (3.7)	
**Malignancy origin: Rectum**	No	12.6 (6)	0.720
	Yes	11.5 (4.8)	
**Obstruction point: Ascending colon**	No	12.5 (6.1)	0.948
	Yes	11.7 (3.6)	
**Obstruction point: Sigmoid colon**	No	12.6 (6.2)	0.966
	Yes	12 (4.9)	
**Obstruction point: Rectum**	No	12.6 (6)	0.812
	Yes	11.6 (4.8)	
**Operation: Colectomy + anastomosis**	No	12.3 (5.3)	0.981
	Yes	12.5 (6.2)	
**Operation: Hartmann’s procedure**	No	12.5 (6.4)	0.711
	Yes	12.1 (3.1)	
**Operation: Stoma**	No	13 (5.4)	**0.040**
	Yes	10.2 (6.7)	
**Operation: Intestinal resection**	No	12.1 (5.7)	0.275
	Yes	14.4 (5.9)	
	**Correlation coefficient for length of stay**		
**Age**	−0.15		0.201
**Preoperative waiting interval**	0.50		**<0.001**
**No of harvested lymph nodes**	−0.14		0.343
**No of infiltrated lymph nodes**	0.03		0.826
**N-status**	−0.01		0.931

The significant *p*-values are in bold. T, tumor; M, metastasis.

**Table 5:** Univariable analysis of patients’ characteristics, perioperative details and histopathological assessment with ICU admission after malignant bowel obstruction surgery.

		ICU Admission				*p*-Value
		No		Yes		
		N	%	N	%	
**Type of hospital**	University	19	61.3	12	38.7	**<0.001**
	Rural	39	100.0	0	0.0	
**Age, mean (SD)**		76.1 (10.8)		76.1 (10)		0.991
**Gender**	Male	29	80.6	7	19.4	0.599
	Female	29	85.3	5	14.7	
**Comorbidities**	No	26	68.4	12	31.6	**<0.001**
	Yes	32	100.0	0	0.0	
**Previous surgery**	No	41	83.7	8	16.3	0.743
	Yes	17	81.0	4	19.0	
**Malignancy status**	Recurrence	10	58.8	7	41.2	**0.006**
	Initial presentation	48	90.6	5	9.4	
**Previously known metastatic lesion at obstruction site**	Yes	14	63.6	8	36.4	**0.007**
	No	44	91.7	4	8.3	
**Macroscopically visible foci**	Yes	27	79.4	7	20.6	0.479
	No	25	86.2	4	13.8	
**Differentiation**	Low	18	85.7	3	14.3	0.641
	Moderate/High	25	92.6	2	7.4	
**No of harvested lymph nodes, mean (SD)**		18.5 (10)		19.2 (7.9)		0.614
**No of infiltrated lymph nodes, mean (SD)**		3.2 (5.6)		0.8 (1.2)		0.358
**Perineural or perivascular infiltrations**	Yes	36	87.8	5	12.2	0.267
	No	5	71.4	2	28.6	
**Τ**	Τis/T1/T2/T3	23	88.5	3	11.5	1.000
	T4	17	85.0	3	15.0	
**Ν status, mean (SD)**		1 (1)		0.4 (0.5)		0.225
**Μ**	No	16	100.0	0	0.0	1.000
	Yes	23	95.8	1	4.2	
**Malignancy type: Colorectal cancer**	No	4	50.0	4	50.0	**0.025**
	Yes	54	87.1	8	12.9	
**Malignancy origin: Cecum**	No	53	81.5	12	18.5	0.579
	Yes	5	100.0	0	0.0	
**Malignancy origin: Ascending colon**	No	51	83.6	10	16.4	0.646
	Yes	7	77.8	2	22.2	
**Malignancy origin: Rectum**	No	43	78.2	12	21.8	0.057
	Yes	15	100.0	0	0.0	
**Obstruction point: Ascending colon**	No	49	83.1	10	16.9	1.000
	Yes	9	81.8	2	18.2	
**Obstruction point: Sigmoid colon**	No	32	76.2	10	23.8	0.106
	Yes	26	92.9	2	7.1	
**Obstruction point: Rectum**	No	42	77.8	12	22.2	0.055
	Yes	16	100.0	0	0.0	
**Preoperative waiting interval (days), mean (SD)**		2.4 (2.7)		5.1 (3.5)		**0.002**
**Operation: Colectomy + anastomosis**	No	29	82.9	6	17.1	1.000
	Yes	29	82.9	6	17.1	
**Operation: Hartmann’s procedure**	No	41	78.8	11	21.2	0.166
	Yes	17	94.4	1	5.6	
**Operation: Stoma**	No	47	85.5	8	14.5	0.271
	Yes	11	73.3	4	26.7	
**Operation: Intestinal resection**	No	52	83.9	10	16.1	0.618
	Yes	6	75.0	2	25.0	

The significant *p*-values are in bold. ICU, Intensive Care Unit; T, tumor; M, metastasis.

**Table 6:** Multivariable analysis of patients’ characteristics, perioperative details and histopathological assessment with postoperative outcomes after malignant bowel obstruction surgery.

Parameters		Postoperative Outcome	*p*-Value
		**30-Day Mortality**	
**Age**		OR 1.21 (95% CI 1.07–1.37)	**0.002**
**Macroscopically visible foci**	No		**0.005**
	Yes	OR 49.61 (95% CI 3.25–758.06)	
**Obstruction point: Sigmoid colon**	No		**0.012**
	Yes	OR 0.03 (95% CI 0.002–0.47)	
		**Length of stay**	
**Operation: Stoma**	No		**0.004**
	Yes	β −0.19SE 0.06	
		**ICU admission**	
**Malignancy status**	Recurrence		**0.005**
	Initial presentation	OR 0.15 (95% CI 0.04–0.57)	

The significant *p*-values are in bold.

## Data Availability

The data that support the findings of this study are available from the corresponding author upon reasonable request.

## Supplementary Materials

The following supporting information can be downloaded at: https://www.mdpi.com/article/10.3390/jcm13010263/s1, Table S1: The comorbidities of patients that underwent surgery due to malignant bowel obstruction; Table S2: The surgical history of patients that underwent surgery due to malignant bowel obstruction; Table S3: Histopathological characteristics of specimens after malignant bowel obstruction; Table S4: The primary malignancy type of tumors causing malignant bowel obstruction.

## References

[1] Mármol I., Sánchez-de-Diego C., Pradilla Dieste A., Cerrada E., Rodriguez Yoldi M.J. (2017). Colorectal carcinoma: A general overview and future perspectives in colorectal cancer. Int. J. Mol. Sci..

[2] Pujara D., Chiang Y.-J., Cormier J.N., Bruera E., Badgwell B. (2017). Selective approach for patients with advanced malignancy and gastrointestinal obstruction. J. Am. Coll. Surg..

[3] Badgwell B.D., Smith K., Liu P., Bruera E., Curley S.A., Cormier J.N. (2009). Indicators of surgery and survival in oncology inpatients requiring surgical evaluation for palliation. Support. Care Cancer.

[4] Anthony T., Baron T., Mercadante S., Green S., Chi D., Cunningham J., Herbst A., Smart E., Krouse R.S. (2007). Report of the clinical protocol committee: Development of randomized trials for malignant bowel obstruction. J. Pain Symptom Manag..

[5] Furnes B., Svensen R., Helland H., Ovrebo K. (2016). Challenges and outcome of surgery for bowel obstruction in women with gynaecologic cancer. Int. J. Surg..

[6] Ripamonti C.I., Easson A.M., Gerdes H. (2008). Management of malignant bowel obstruction. Eur. J. Cancer.

[7] Shariat-Madar B., Jayakrishnan T.T., Gamblin T.C., Turaga K.K. (2014). Surgical management of bowel obstruction in patients with peritoneal carcinomatosis. J. Surg. Oncol..

[8] Cousins S.E., Tempest E., Feuer D.J. (2016). Surgery for the resolution of symptoms in malignant bowel obstruction in advanced gynaecological and gastrointestinal cancer. Cochrane Database Syst. Rev..

[9] Fackche N.T., Johnston F.M. (2021). Malignant Bowel Obstruction. Adv Surg.

[10] Winner M., Mooney S.J., Hershman D.L., Feingold D.L., Allendorf J.D., Wright J.D., Neugut A.I. (2013). Management and outcomes of bowel obstruction in patients with stage IV colon cancer: A population-based cohort study. Dis. Colon Rectum.

[11] Krouse R.S. (2019). Malignant bowel obstruction. J. Surg. Oncol..

[12] Shariff F., Bogach J., Guidolin K., Nadler A. (2022). Malignant Bowel Obstruction Management Over Time: Are We Doing Anything New? A Current Narrative Review. Ann. Surg. Oncol..

[13] Winner M., Mooney S.J., Hershman D.L., Feingold D.L., Allendorf J.D., Wright J.D., Neugut A.I. (2013). Incidence and predictors of bowel obstruction in elderly patients with stage IV colon cancer: A population-based cohort study. JAMA Surg..

[14] Boeding J.R., Ramphal W., Crolla R.M., Boonman-de Winter L.J., Gobardhan P.D., Schreinemakers J.M. (2018). Ileus caused by obstructing colorectal cancer—Impact on long-term survival. Int. J. Color. Dis..

[15] Franke A.J., Iqbal A., Starr J.S., Nair R.M., George T.J. (2017). Management of Malignant Bowel Obstruction Associated with GI Cancers. J. Oncol. Prac..

[16] Wong S.L. (2009). Lymph node counts and survival rates after resection for colon and rectal cancer. Gastrointest. Cancer Res..

[17] Vogel J.D., Eskicioglu C., Weiser M.R., Feingold D.L., Steele S.R. (2017). The American Society of Colon and Rectal Surgeons Clinical Practice Guidelines for the Treatment of Colon Cancer. Dis. Colon Rectum.

[18] Madariaga A., Lau J., Ghoshal A., Dzierżanowski T., Larkin P., Sobocki J., Dickman A., Furness K., Fazelzad R., Crawford G.B. (2022). MASCC multidisciplinary evidence-based recommendations for the management of malignant bowel obstruction in advanced cancer. Support. Care Cancer.

[19] Käser S.A., Mattiello D., Maurer C.A. (2016). Distant Metastasis in Colorectal Cancer is a Risk Factor for Anastomotic Leakage. Ann. Surg. Oncol..

[20] Krouse R.S., Anderson G.L., Arnold K.B., Thomson C.A., Nfonsam V.N., Al-Kasspooles M.F., Walker J.L., Sun V., Alvarez Secord A., Han E.S. (2023). Surgical versus non-surgical management for patients with malignant bowel obstruction (S1316): A pragmatic comparative effectiveness trial. Lancet Gastroenterol. Hepatol..

[21] Harris G.J., Senagore A.J., Lavery I.C., Fazio V.W. (2001). The management of neoplastic colorectal obstruction with colonic endolumenal stenting devices. Am. J. Surg..

[22] Currow D.C., Quinn S., Agar M., Fazekas B., Hardy J., McCaffrey N., Eckermann S., Abernethy A.P., Clark K. (2015). Double-blind, placebo-controlled, randomized trial of octreotide in malignant bowel obstruction. J. Pain Symptom Manag..

