# Supplementary material for: Malignant Bowel Obstruction: A Retrospective Multicenter Cohort Study
Source: J Clin Med. 2024 Jan 2;13(1):263. doi: 10.3390/jcm13010263 (PMC10779546; doi:10.3390/jcm13010263)
Supplement: Supplementary file 1 [file jcm-13-00263-s001.zip › jcm-2771420-supplementary.pdf]

**Supplementary Table S1. Patient Comorbidities**		
**Disease**	**N**	**%**
Hypertension	26	37,1
Coronary Disease	14	20
Hypothyroidism	12	17,1
Dyslipidemia	8	11,4
Heart arrhythmia	7	10
Non-GI malignancy	7	10
Diabetes	7	10
Prostate hyperplasia	5	7,1
Abdominal aortic aneurysm	3	4,3
Heart valve abnormality	2	2,9
COPD	2	2,9
Rheumatoid arthritis	2	2,9

Supplementary Table S1. The comorbidities of patients that underwent surgery due to malignant bowel obstruction.

Supplementary Table S2. Surgical History		
**Surgical Operation**	**N**	**%**
Appendectomy	9	12,9
Inguinal hernia repair	7	10
Laparoscopic Cholecystectomy	6	8,6
Uterus resection	4	5,7
Hip arthroplasty	3	4,3
Left hemicolectomy	2	2,9
Intestinal resection	2	2,9
Right extended colectomy	2	2,9
Mastectomy	2	2,9
Lumpectomy	2	2,9
Prostatectomy	2	2,9
Hartmann's procedure	1	1,4
Abdominal aortic aneurysm repair	1	1,4
Distal Pancreatectomy	1	1,4
Subtotal gastrectomy	1	1,4
Total gastrectomy	1	1,4
Sigmoidectomy	1	1,4
Splenectomy	1	1,4
Abdominoperineal resection	1	1,4
Esophageal perforation	1	1,4
Low anterior resection	1	1,4

Supplementary Table S2. The surgical history of patients that underwent surgery due to malignant bowel obstruction.

Supplementary Table S3. Histopathological assessment			
		**N**	**%**
**Malignancy type**	Colon adenocarcinoma	59	89,4
	Pancreatic adenocarcinoma	2	3,0
	Gastric adenocarcinoma	2	3,0
	Neuroendocrine tumor (NET)	2	3,0
	Lymphoma	1	1,5
**Differentiation**	Low	21	43,8
	Moderate	24	50,0
	High	2	4,2
	In situ	1	2,1
**Margins**	Free	44	89,8
	Infiltrated	5	10,2
**Perineural or perivascular infiltrations**	Yes	41	85,4
	No	7	14,6
**T (Tumor)**	Tis	2	4,3
	T1	0	0,0
	T2	2	4,3
	T3	22	47,8
	T4	20	43,5
**N (Node)**	0	20	44,4
	1	11	24,4
	2	12	26,7
	3	2	4,4
**M (Metastasis)**	No	16	40,0
	Yes	24	60,0
**No of harvested lymph nodes, mean (SD)**		18,6 (9,7)	
**No of infiltrated lymph nodes, mean (SD)**		2,9 (5,3)	

Supplementary Table S3. Histopathological characteristics of specimens after malignant bowel obstruction.

**Supplementary Table S4. Malignancy origin**		
**Organ**	**N**	**%**
Sigmoid colon	30	42,9
Rectum	15	21,4
Ascending colon	9	12,9
Cecum	5	7,1
Transverse colon	5	7,1
Descending colon	5	7,1
Other than intestine	5	7,1
Small intestine	2	2,9

Supplementary Table S4. The primary malignancy type of tumors causing malignant bowel obstruction.
